# Glucocorticoids, Sex Hormones, and Immunity

**DOI:** 10.3389/fimmu.2018.01332

**Published:** 2018-06-12

**Authors:** Oxana Bereshchenko, Stefano Bruscoli, Carlo Riccardi

**Affiliations:** ^1^Section of Pharmacology, Department of Medicine, University of Perugia, Perugia, Italy; ^2^Department of Surgery and Biomedical Sciences, University of Perugia, Perugia, Italy

**Keywords:** glucocorticoids, sex hormones, innate immunity, adaptive immunity, glucocorticoid receptor, estrogen receptor alpha

## Abstract

Glucocorticoid hormones regulate essential body functions in mammals, control cell metabolism, growth, differentiation, and apoptosis. Importantly, they are potent suppressors of inflammation, and multiple immune-modulatory mechanisms involving leukocyte apoptosis, differentiation, and cytokine production have been described. Due to their potent anti-inflammatory and immune-suppressive activity, synthetic glucocorticoids (GCs) are the most prescribed drugs used for treatment of autoimmune and inflammatory diseases. It is long been noted that males and females exhibit differences in the prevalence in several autoimmune diseases (AD). This can be due to the role of sexual hormones in regulation of the immune responses, acting through their endogenous nuclear receptors to mediate gene expression and generate unique gender-specific cellular environments. Given the fact that GCs are the primary physiological anti-inflammatory hormones, and that sex hormones may also exert immune-modulatory functions, the link between GCs and sex hormones may exist. Understanding the nature of this possible crosstalk is important to unravel the reason of sexual disparity in AD and to carefully prescribe these drugs for the treatment of inflammatory diseases. In this review, we discuss similarities and differences between the effects of sex hormones and GCs on the immune system, to highlight possible axes of functional interaction.

## Introduction

The interaction between endocrine and immune systems ensures the correct function of immune system. Women mount stronger immune responses against foreign but also against self-antigens, and the prevalence of most autoimmune diseases (AD) is greatly increased in women compared to men (1–4). An important role underlying the difference in activity of immune cells in men and women is attributed to sex hormones (4, 5).

Steroid hormones, such as estrogens, prolactin, progesterone, and glucocorticoids (GCs) modulate the development and activity of both innate and adaptive immunity differently in men and women (2, 5–8). Therefore, characterization of the mechanisms of hormonal regulation of different immune cell types is important for understanding the regulatory circuits critical for keeping a competent and a healthy immune system and to improve therapy of AD.

The degree and the duration of the immune response is influenced by the number and the type of circulating immune cells; therefore, the effect of steroid hormones on survival and differentiation of T and B lymphocytes and cells of innate immune system will define the numeric leukocyte output in the periphery. Hormonal regulation of cytokine production impacts on differentiation of naïve T cells into the particular effector subtypes, thus defining the type of the mounted immune responses. Interestingly, since sex hormones and GCs are acting on the same cellular pathways that regulate leukocytes growth, differentiation, and survival, their simultaneous action would likely enhance or abrogate the effects elicited by individual factors. Therefore, biologic differences in endogenous GCs levels, as well as exposure of an individual to specific environmental stimuli, including presence of chronic inflammatory disease, prolonged stress, metabolic challenges or injuries, as well as pharmacologic administration of exogenous GCs, will alter the expected gender-related effects on immunity and ADs. The converging and diverging effects of GCs and sex hormones on different cells types of the immune system are discussed in this minireview.

## Steroid Hormones: Mechanisms of Action

Corticosteroids and sex hormones are derived from cholesterol through the same sterodoigenic pathway, with common metabolic intermediate, progesterone, and are under the control of the hypothalamus–pituitary–adrenal gland (HPA) axis (9). The main natural GCs (i.e., cortisol) are produced in the cortical part of the adrenal gland (9). Biosynthesis of androgens, including testosterone, occurs mainly in Leydig cells in male gonads, and small amounts are produced by the ovary and adrenal cortex in females (9). The androgens dehydroepiandrosterone (DHEA), androstenedione, and testosterone are the precursors of estrogens, produced in females primarily in ovaries (9).

### Glucocorticoids

Glucocorticoids are essential endocrine regulators of body functions in homeostasis and adaptation to environmental changes. One important feature of GCs regulation is the circadian control of GCs secretion by the HPA axis. The rhythmically released GCs may have an impact on immunity regulation (10). Endogenous GCs act on a variety of cell types to regulate the expression of genes controlling cellular metabolism, growth, differentiation, and apoptosis (11, 12). Thus, proper production and activity of the endogenous GCs is critical for the regulation of inflammatory events during tissue repair and pathogens elimination. Due to their potent immune-suppressive and anti-inflammatory function, synthetic GCs are extensively used in clinic to treat acute and chronic inflammation (13, 14).

The GCs act *via* genomic (transcriptional) and non-genomic (transcription-independent) mechanisms. Most cellular actions of GCs are mediated by binding to its cognate intracellular receptor (GR), transcription factor of the nuclear receptors (NR) superfamily (15). GR shares functional domains with other NR that include an N-terminal transactivation domain, a highly conserved central DNA binding domain, and a C-terminal ligand-binding domain (11). After ligand-induced conformational changes, GR translocates into the nucleus where it regulates transcription of hundreds of genes. It may bind directly to DNA *via* glucocorticoid recognition elements, or regulate gene expression *via* indirect mechanisms (16, 17). GR may directly interact with NF-κB (17, 18), a key transcription factor that activates many pro-inflammatory genes (19), as well as with other transcription factors (TFs), such as STAT-3 and -5 (20, 21), AP-1 (22), and CREB (23). GR does not interfere with the DNA binding activity of NF-κB, but inhibits its transcriptional activation function *via* preventing its nuclear translocation (17, 18), or interfering with transcriptional machinery by competition with co-factors such as p300/CBP (24), thus repressing the expression of pro-inflammatory genes, such as TNF-α, IL-1, and ICAM-1 (13, 25). Mechanisms of GCs actions also include induction of proteins with anti-inflammatory activities, such as glucocorticoid-induced leucine zipper (GILZ) (26, 27), which mediates many of the GCs’ activities (28–31), including inhibition of RAS/RAF/MAPK pathways (32, 33), and of NF-κB and AP-1 activities (34–37). “Non-genomic” effects of GCs include direct interaction of liganded GR with diverse intracellular mediators and modulating several signaling pathways, including protein kinase C, phosphatidylinositol-specific phospholipase C, and src kinase pathways (38–42).

### Sex Hormones

Sex hormones regulate reproductive and metabolic body functions throughout the life of the subjects. Sex hormones influence immune cell function and inflammation: androgens are mainly anti-inflammatory (7), whereas estrogens have both pro- and anti-inflammatory roles, depending on several factors, such as type of immune response or variability of expression of different estrogen receptor (ER) isoforms (8).

Estrogens exert their effects through binding to ERα or ERβ, TFs of NR superfamily that regulate expression of genes involved in cell survival, proliferation, differentiation, and reproductive functions (6, 43). Similar to GR, nuclear ERs bind DNA either directly through estrogen response elements, or indirectly, *via* ERE-independent TFs, such as NF-κB, SP1, AP-1, C/EBPβ (43–45), to induce or repress gene expression. Estrogens also elicit rapid (“non-genomic”) signal transduction effects, *via* modulation of intracellular calcium, cAMP, potassium currents, phospholipase C activation, and stimulation of PI3K/AKT and ERK pathways (44).

Estrogen receptors are expressed in various types of immune cells, including lymphocytes, macrophages, and dendritic cells (DC) (5, 8). Estrogens were shown to exert both, anti- and pro-inflammatory effects, depending on the context and combination of factors that include: the type of the immune cell target, the concentration of the hormone, the type of immune stimulus (foreign or auto-antigens), the target organ microenvironment, and the relative expression of ERα and ERβ. Estrogens may promote inflammation *via* regulation of the expression of inflammatory mediators *via* Akt/mTOR pathway (46, 47). However, pregnancy or higher doses of ectopic estrogens typically suppress immune responses (4), by repressing the expression of multiple NF-κB- and c-Jun-driven cytokine genes (45, 48–50), similar to GCs. ERα may displace p65 and CREB and their associated co-regulators from NF-κB binding site (51). Progesterone (P4) is produced at high levels during the menstrual cycle and during pregnancy. P4 signals through the progesterone receptor (PR) and to a lesser extent, through GR and mineralocorticoid receptors. P4 is expressed in different immune cells types, including NK, macrophages, DCs, and T cells (52), and have broad anti-inflammatory effects of the immune system by decreasing leukocytes activation and production of pro-inflammatory mediators (5). NF-κB inhibition is also suggested to play a role in these effects of P4 (53).

Similar to GCs, male steroid hormones demonstrate mostly the suppressive role in immune function (2, 54–57), *via* binding to androgen receptor (AR), also a member of NR superfamily, and regulation of target gene expression (58). AR recognizes directly the androgen response elements in the regulatory regions of AR target genes (59). Androgens, including dihydrotestosterone and testosterone, generally suppress immune cell activity, by reducing the inflammatory and promoting anti-inflammatory mediators’ expression by macrophages and T cells (5, 60–62). The levels of androgen DHEA are reduced in patients with RA (63) and inflammatory bowel disease (64) suggesting that DHEA may cover many aspects of immune regulatory effects of sex hormones.

Moreover, an indirect immunomodulatory effect of androgens may be related to the anti-inflammatory activity of endogenous GCs due to their effect on the HPA axis (65).

## Modulation of Immune Responses by Steroid Hormones

### Effect of Steroid Hormones on Innate Immunity

Both GCs and sex steroid modulate the development and function of various cells of innate immunity, including neutrophils, macrophages, natural killer cells, and DC (Table 1). GCs actions include regulation of apoptosis in many cell types: they exert a protective effect in macrophages, by inhibiting activation of caspases and contributing to inflammation resolution (66); however, prolonged usage of GCs promotes apoptosis in macrophages (67), natural killer cells (68), DC (69–71), neutrophils (72, 73), and eosinophils (72). To the contrary, estrogens and androgens increase the number of neutrophils (74, 75).

**Table 1:** Effect of steroid hormones on innate and adaptive immunity.

Immune target	Glucocorticoids	Estrogens	Androgens
Macrophages	High dose, activity ↓ (67, 76) Apoptosis ↓↑ (66, 67) Inflamm cytokines ↓ (76)	Inflamm cytokines↓ (94, 95)	Inflamm cytokines ↓ (5)

DC	Apoptosis ↑ (76) Maturation ↓ (100) Inflamm cytokines↓ (101–103) IL-10, TGF-β ↑ (101–103)	Maturation ↑ (107) Inflamm cytokines↑↓ (104–107) IL-10, TGF-β ↑ (109–112)	ND

Neutrophils	Number ↓ (72, 73) Trafficking ↓ (80–82) Apoptosis ↑ (72, 73)	Number ↑ (74, 75) Trafficking ↓ (83–88) Activation ↓ (92, 93) Inflamm cytokines↑↓ (96–99)	Number ↑ (62) Trafficking ↑ (62) Inflamm cytokines↑

Thymocytes	Apoptosis ↑ (38, 39, 113–116) Proliferation ↓ (13)	Proliferation ↓ (123–126)	Proliferation ↓(127, 128)

Th1 cells	Apoptosis ↑ (130) Th1 cytokines ↓ (131–133)	Activation ↓ Th1 cytokines ↑ (149–153) High levels ↓ (155–157)	Activation ↓ Th1 cytokines ↓ (60)

Th2 cells	Apoptosis ↑↓ Th2 cytokines ↑	Th2 cytokines↑ (155–158)	Activation ↓ Th2 cytokines ↓↑ (5, 60)

Th17 cells	Apoptosis ↑↓ (135) Th17 cytokines ↓ (134)	Number ↓ (161) Th17 cytokines ↑↓ (159–161)	Number ↓ Th17 cytokines ↑ (5)

Treg cells	Number ↑ (136–140) Function ↑ (101, 141–143)	Number ↑ (162–166)	Number ↑ (5)

B cells	Apoptosis ↑ (167, 168) Number ↓ Ig ↓↑ (171–173)	Apoptosis↓ (175–177) Ig ↑ (5)	Number ↓ (5, 62) Ig ↓ (5)

ILCs	ILC2 ↓ (180)	Uterine ILC2 ↑ (179)	Lung ILC2 ↓ (181)

*DC, dendritic cells; Th, T helper cells; Treg, regulatory T cells; ILC, innate lymphoid cells; ND, not determined; Ig, immunoglobulins*.

Glucocorticoids have mainly suppressive effects on the cells of innate immunity (Table 1). High doses of GCs inhibit most of the functions of tissue macrophages, such as chemotaxis, phagocytosis, proliferation, and antigen presentation (67, 76). GCs suppress the expression of various pro-inflammatory cytokines released by macrophages, such as IFN-γ, IL-1α, and IL-1β (76). In addition, the synthesis of anti-inflammatory molecules annexin A1 and GILZ in macrophages contributes to the anti-inflammatory and immunosuppressive action of GCs (28, 29, 77). GCs also inhibit monocytes’ chemotaxis by reducing expression of chemokines, such as CXCL-1, IL-8 and CXCL-2, and CCL2 (78, 79), control granulocyte trafficking by reducing expression of adhesion molecules, such as Mac-1 and L-selectin on neutrophils (80–82), thus limiting the inflammatory response. In addition, GCs prevent neutrophils migration into inflamed tissues *via* the upregulation of GILZ and annexin A1 (82). Similar to GCs, treatment with estrogens inhibits neutrophils’ activity by restricting their recruitment (83–88) and inhibiting the NFκB-dependent production of major neutrophil chemoattractants CXCL-1, CXCL2, CXCL3, and CXCL8 in experimental models of tissue injury (86–91). Estradiol inhibits neutrophil activation through a reduction in oxidative metabolism (92), adhesion to endothelial cells *via* upregulation of the anti-inflammatory protein annexin A1 (93), and attenuates the release of pro-inflammatory cytokines, such as TNF-α, IL-1β, and IL-6 in human peripheral blood mononuclear cells (94, 95) and in neutrophils and macrophages (96–99). Interestingly, the inhibition of NFκB activity is a central mechanism underlying these actions (83).

Glucocorticoids and estrogens have both convergent and divergent actions in DCs. GCs inhibit DC function in several ways: by promoting apoptosis (76), disturbing maturation of immature DCs (100), and inducing a tolerogenic phenotype, *via* downregulating the expression of major histocompatibility complex (MHC)-II and co-stimulatory molecules and cytokines, such as IL-1, IL-6, and IL-12 (101, 102). Such changes are associated with reduced proliferative and T helper 1 (Th1) responses by T cells (103), and increase in immunosuppressive regulatory T (Treg) cells (101). Instead, estrogens promote DC cell differentiation and MHCII expression, and induce the expression of IL-6, IL-23, IL-12, and IL-1β (104–107), thus increasing the type-1 responses (108). On the other hand, similar to GCs, estrogens induce a tolerogenic phenotype in DC, by decreasing the expression of pro-inflammatory cytokines and chemokines, such as IL-6, IFN-γ, IL-12, CXCL8, and CCL2 (109–111), and upregulating inhibitory molecules PD-L1 and PD-L2, and regulatory cytokines IL-10 and TGF-β, thus also leading to a decrease in the Th1 cells activation and a shift toward production of Th2-type cytokines (109, 112).

### Effect of Steroid Hormones on Adaptive Immunity

Controlled elimination of T cells during thymocyte development and T cell-mediated immune responses is essential to prevent immunopathologies, such as autoimmunity and cancer. GCs induce apoptosis in developing thymocytes (113–116) and regulate both “death by neglect” and positive selection (117). The GC-induced apoptosis in thymocytes is also attributed to non-genomic effects of GR (38, 39, 118, 119). Their role in positive selection is inferred from the antagonism between GCs and T cell repertoire (TCR)-activated signals, which allows cells with intermediate TCR affinity to be positively selected (120–122).

The growth suppressive effect of GCs on thymocytes is common to the action of female and male sex hormones. Similar to GCs, estrogens inhibit thymocyte proliferation (123) and induce thymic atrophy (124). Pregnancy is associated with accelerated thymic involution (125, 126). Androgens also restrain active cell cycling and the number of immature thymocytes (127). The number of CD4+ and CD8+ T cells is lower in males around 50–75 of age compared to females, and the diversity of the TCR in females is larger than in males of the same age (128).

Upon TCR activation and stimulation with particular cytokines, naïve mature CD4+ T cells differentiate in the periphery into one of several lineages of T helper (Th) cells that include Th1, Th2, Th17, and Treg cells (129). GCs promote the shift from Th1 to Th2 type immune responses by differentially regulating apoptosis of Th1 and Th2 cells (130), and by interfering with the activity of their master regulators T-bet and GATA-3, respectively (131–133). GCs can also suppress the production of TNF-α, IL-12, and IFN-γ and induce the production of IL-4, IL-10, and IL-13 (13, 130). GCs inhibit the production of Th17-type cytokines in AD (134), although the sensitivity of Th17 cells to GC-induced apoptosis varies dependent on disease-specific microenvironment (135). Treg cells play a critical role in regulating immune responses and peripheral tolerance. GCs upregulate expression of FoxP3, the master regulator of Treg cells, expand Treg cell population (136–140), and increase Treg function in AD (141–143). Expression of GCs’ target gene GILZ also contributes to the GC-mediated regulation of Th1/Th2 balance (144, 145), and the induction of Treg cells by promoting TGF-β-dependent FoxP3 expression (136).

Sex steroids also modulate the differentiation and function of all subsets of T cells (75, 146–148). Contrary to GCs, estrogens promote INF-γ production by Th1 cells in both human and mice (149–151), via potentially direct interaction of ER with the *Ifng* promoter (150, 152), upregulation of Th-1 transcription factor T-bet (151, 153), or via microRNA-dependent suppression of IFN-γ expression (154). However, high levels of estrogen skew the immune response from Th1 to Th2-type (155–157), similar to GCs. Estradiol also regulates anti-inflammatory Th2 shift by activating SGK1 kinase (158). The effects of estrogens on Th17 subset are different depending on experimental model, leading to enhancement (159, 160) or decrease (161) of Th17-dependent inflammation. Like GCs, estrogens promote the expansion of Treg cells (162, 163) by upregulating the expression of FoxP3, PD-1, and CTLA-4 (162–166), therefore, GCs and estrogens may co-operate in promoting the Treg development and activity.

Glucocorticoids and estrogens elicit opposite effects on B cells. GCs have a pro-apoptotic effect on developing B lymphocytes in the bone marrow (167, 168). On the other hand, B-lymphoblastic leukemia cells are resistant to GC-induced apoptosis, due to enhanced expression of B-cell lymphoma-2 protein (167, 169). GILZ mediates GC-induced apoptosis in B cells as shown by the accumulation of B cells in the bone marrow and in the periphery in GILZ-deficient mice, due to reduced B cell apoptosis (170). GCs affect directly humoral response by reducing circulating immunoglobulins (Igs) although some studies have shown an increase of IgE production in conjunction with IL-4 (171–173). Instead, enhanced antibody production is observed in women, suggesting that female sex hormones stimulate B cell-mediated responses. Estrogen treatment also interferes with normal tolerance of naive DNA-reactive B cells, thus contributing to the development of AD. Elevated estrogen alters the negative selection of DNA-reactive B cells in the periphery (174, 175), interfering with proper B cell receptor signaling and regulation of B cells activation and apoptosis (176, 177). Thus, pharmacologic treatment with synthetic GCs may be useful in suppression of the enhanced B cell activities in AD.

### Effect of Steroid Hormones on Innate Lymphoid Cells (ILC)

Innate lymphoid cell is a most recently identified immune cell type, which contributes to inflammation, immunity, and the maintenance of tissue integrity and homeostasis (178). Recent evidence demonstrated that group 2 ILC2s are present in the uterus under control of estrogens and are increased upon estrogen administration (179). It is, therefore, possible that estrogens modulate tolerance *via* ILC2-mediated modulation of the protective Th2 shift in pregnancy. Instead, ILC2 promote lung inflammation during asthma (180). Interestingly, male mice have reduced numbers of ILC2s in peripheral tissues compared to females, and the number of ILC2s in the lungs is negatively regulated by androgens (181), consistent with the overall suppressive role of male steroids in immunity. GCs were shown to modulate the cytokine production by the ILC2s, thus these finding suggests a modulatory role of steroid hormones in ILCs and homeostasis of specific tissues.

## Interaction Between GR and Sex Hormone Receptors

GR, ER, and AR are ligand-activated TFs belonging to the NR superfamily (182). Experimental evidence shows that GR and sex hormone receptors share some common mechanisms of gene regulation, but they also exploit different mechanisms to repress pro-inflammatory genes depending on the target gene, cell type, and interactions with other TFs.

Estrogen receptor, AR, and GR induce expression of genes that control proliferation, differentiation, and cell death by directly binding to specific hormone response elements or by indirectly tethering through TFs, such as AP1 (22, 183–185), Sp1 (186, 187), Stat1 (188), and NFκB (189, 190). The potential crosstalk in the regulation of gene expression by GR and ER was studied mostly in non-immune cell types, which may, however, provide mechanistic evidence of the mechanisms potentially operating in cells of the immune system.

The functional interaction between GR and ER signaling has been observed in several cancer cell lines, and requires additional factors, such as MED14, SRC-2, and SRC-3 in the same complex, resulting in either cooperative or mutually inhibiting effects (191). GR may inhibit the action of ER *via* distinct mechanisms. GCs may inhibit the estrogen signaling indirectly, by inducing the estrogen-metabolizing enzyme in breast cancer cells (192). Synthetic GC dexamethasone (Dex) antagonizes ERα-regulated target gene expression in breast cancer cells treated with estrogen and Dex simultaneously *via* the direct protein–protein interaction and the recruitment of GR to ERα binding sites (193). This recruitment is facilitated by AP-1 and leads to a displacement of ERα from DNA and repression of its target genes transcription (193, 194).

Reciprocally, the ER-mediated inhibition of the GR function was also described. Treatment of the breast cancer cell line with estrogen agonists downregulates GR protein levels *via* proteosome-mediated degradation (195). On the other hand, in an experimental lung inflammation rat model, the ER antagonist (ICI 182780) blocked the anti-inflammatory effects of GCs, suggesting that GR and ER co-operate in this setting in their anti-inflammatory activity (196). A subset of pro-inflammatory genes was repressed by both ER and GR (CD69, MCP-1, IL-6, IL-8), and the ER antagonist blocked Dex-mediated repression of these genes (197), by preventing the recruitment of nuclear coactivator 2 by the GR necessary for trans-repression. These data suggest that GR and ER functionally cooperate on selected promoters.

The functional effect of the interaction of GR with PR and ER is less characterized. PR and GR were shown to interact *in vitro*, and *in vivo*. Progesterone acts *via* GR to repress IL-1β-driven COX-2 activation (198, 199). The AR and the GR form heterodimers at a common DNA site both *in vitro* and *in vivo*, and this interaction leads to mutual inhibition of transcriptional activity (200).

## Conclusions

Most of the mechanistic insights into synergistic and antagonizing effects of GR and sex steroid receptors in gene expression were obtained in non-immune cells, and, to our knowledge, the interactions between GCs and sex hormones in immune cells have not been studied *in vitro*. However, receptors for both classes of hormones are present in variety of immune cells, where, as reviewed above, they have been separately shown to influence various aspect of immune cell activity, ranging from cell survival to differentiation and expression of pro- and anti-inflammatory molecules (Table 1). Thus, the investigation of possible mutual influence of GCs and sex hormones in immune cells and its mechanisms is warranted.

The effects of female sex hormones on cells of adaptive immune system such as Th cell differentiation and B cells may underlie the higher predisposition of women to AD (4). The GC-mediated suppression of Th1 and promotion of Treg cell activity, as well as apoptotic effects on B cells may explain in part the cellular basis of GCs’ efficacy in dampening the symptoms of many AD (13). Development of novel therapies for immune cell type- and gender-specific modulation of immune system may represent future direction for treatment of AD.

## Author Contributions

OB, SB, and CR wrote and edited the manuscript.

## Conflict of Interest Statement

The authors declare that the research was conducted in the absence of any commercial or financial relationships that could be construed as a potential conflict of interest.
